# A High-Resolution 3D Ultrasound Imaging System Oriented towards a Specific Application in Breast Cancer Detection Based on a 1 × 256 Ring Array

**DOI:** 10.3390/mi15020209

**Published:** 2024-01-30

**Authors:** Junhui Zhang, Fei Wu, Fansheng Meng, Guojun Zhang, Renxin Wang, Yuhua Yang, Jiangong Cui, Changde He, Licheng Jia, Wendong Zhang

**Affiliations:** 1 State Key Laboratory of Instrumentation Science and Dynamic Measurement Technology, North University of China, Taiyuan 030051, China; 17835193260@163.com (J.Z.); wufeikitty0311@163.com (F.W.); sz202206139@st.nuc.edu.cn (F.M.); zhangguojun1977@nuc.edu.cn (G.Z.); wangrenxin@pku.edu.cn (R.W.); yangyuhua407@163.com (Y.Y.); jgcui@nuc.edu.cn (J.C.); hechangde@foxmail.com (C.H.); 2 National Key Laboratory for Electronic Measurement Technology, School of Instrument and Electronics, North University of China, Taiyuan 030051, China

**Keywords:** 3D ultrasound imaging system, ultrasonic tomography, ring array, breast imaging, high-resolution

## Abstract

This paper presents the design and development of a high-resolution 3D ultrasound imaging system based on a 1 × 256 piezoelectric ring array, achieving an accuracy of 0.1 mm in both ascending and descending modes. The system achieves an imaging spatial resolution of approximately 0.78 mm. A 256 × 32 cylindrical sensor array and a digital phantom of breast tissue were constructed using the k-Wave toolbox. The signal is acquired layer by layer using 3D acoustic time-domain simulation, resulting in the collection of data from each of the 32 layers. The 1 × 256 ring array moves on a vertical trajectory from the chest wall to the nipple at a constant speed. A data set was collected at intervals of 1.5 mm, resulting in a total of 32 data sets. Surface rendering and volume rendering algorithms were used to reconstruct 3D ultrasound images from the volume data obtained via simulation so that the smallest simulated reconstructed lesion had a diameter of 0.3 mm. The reconstructed three-dimensional image derived from the experimental data exhibits the contour of the breast model along with its internal mass. Reconstructable dimensions can be achieved up to approximately 0.78 mm. The feasibility of applying the system to 3D breast ultrasound imaging has been demonstrated, demonstrating its attributes of resolution, precision, and exceptional efficiency.

## 1. Introduction

According to the latest global cancer statistics, breast cancer has emerged as the most prevalent form of malignancy worldwide [1]. The early detection of breast cancer primarily relies on X-ray mammography, magnetic resonance imaging (MRI), and ultrasound computed tomography [2,3]. The ultrasound technique has emerged as a pivotal modality for breast cancer diagnosis owing to its cost-effectiveness and non-ionizing radiation properties [4,5]. Although widely utilized for visualizing two-dimensional cross-sections of tissues and organs, traditional B-mode ultrasound imaging exhibits limitations in terms of accuracy and intuitiveness [6]. This poses challenges in meeting the precision requirements of medical applications [7,8]. Compared to two-dimensional ultrasound imaging technology, the distinct advantage of three-dimensional ultrasound imaging lies in its ability to depict the internal structure of breast tissue from multiple perspectives. The technique allows for the acquisition of comprehensive information regarding the stereoscopic architecture and pathological characteristics of tissues and organs [9]. Consequently, the investigation of three-dimensional imaging has emerged as a persistent focal point in the field of ultrasound imaging [10]. The concept of three-dimensional ultrasound imaging was initially proposed by Buan and Greewood in 1961 [11], who achieved a three-dimensional representation of a human organ by integrating a series of parallel two-dimensional ultrasonic cross-section images. Consequently, an increasing number of researchers have conducted extensive investigations into the methods of acquiring 3D ultrasound data and techniques for reconstructing three-dimensional structures [12].

In recent years, research teams worldwide have conducted extensive investigations on ultrasound computed tomography devices for breast screening imaging in women, resulting in significant advancements [13]. The development of ultrasonic computed tomography systems based on linear sensor arrays, ring sensor arrays, curved surface sensor arrays, cylindrical sensor arrays, and semi-ellipsoid sensor arrays has been pursued [14,15]. A Computerized Volumetric Ultrasound System (CVUS) was developed by Michael Andre, Steven Johnson et al. (TechniScan, Inc., Salt Lake City, UT, USA) [16,17]. The system comprises of a circular water tank with the tri-arm containing the linear transmitter array, the linear receiver array, and the three linear reflection arrays. The detection capability of linear arrays in the depth direction is limited, resulting in a potential loss of information. Furthermore, artefacts may arise due to target or probe movement. The group of Nebojsa Duric and others (Karmanos Cancer Institute, Detroit, MI, USA) has developed a Computer Ultrasonic Risk Evaluation (CURE) system [18], which is based on the principles of ultrasonic tomography. This system utilizes a ring transducer consisting of 256 equally spaced units. CURE obtains breast volume data by vertical movement in the upward and downward directions. In recent decades, the Softvue system, developed by Delphinus Medical Technologies in the United States [19], has been utilized for diagnosing dense breasts in women and is equipped with a sophisticated array of highly focused sensors. Professor Ming Yue Ding’s team has developed a new USCT research system called Ultralucid [20], which is equipped with a 2048-element ring transducer, 1024 transmit (TX) channels, 1024 receive (RX) channels, two servers, and a control unit. The system demonstrates exceptional scalability and flexibility. The Karlsruhe Institute of Technology (KIT) in Germany has developed two generations of fully three-dimensional ultrasound computed tomography (3D USCT) systems for the purpose of early breast cancer diagnosis through 3D imaging [21]. The first-generation fully three-dimensional ultrasonic computed tomography (3D USCT) system comprises a cylindrical aperture with dimensions of 18 cm in diameter and 15 cm in height, which houses a total of 1920 sensors. The second-generation fully three-dimensional ultrasonic computed tomography (3D USCT) system is equipped with 628 ultrasonic transmitters and 1413 receivers, which are divided into 157 sensor array systems mounted on the inner surface of the measuring basin [22]. The 3D USCT system, which acquires comprehensive breast volume data for reconstruction purposes, necessitates a substantial number of small-sized sensors to achieve the necessary resolution [23], posing a technological challenge in current circumstances. The computational complexity of the 3D USCT system exceeds that of its 2D counterpart, requiring more advanced algorithms for three-dimensional reconstruction [24]. Due to the extensive volume of collected data, there are persistent bottlenecks, such as inadequate memory capacity and sluggish data retrieval speed, which impede the precision of imaging data [25]. The two-dimensional ring array exhibits enhanced omnidirectional detection capability and can achieve improved spatial resolution by increasing the number of sensor arrays [26,27]. Therefore, the utilization of a two-dimensional ring array for three-dimensional ultrasonic image reconstruction can streamline the data acquisition and processing procedures [28], improve imaging speed, and facilitate real-time imaging.

The utilization of three-dimensional imaging surpasses that of two-dimensional imaging in terms of acquiring comprehensive information, thereby proving invaluable for the accurate diagnosis and precise localization of lesions. Therefore, in order to obtain more comprehensive imaging results, a 3D ultrasonic imaging system based on a 1 × 256 ring array is designed and built in this paper. A 3D simulation dataset was obtained by establishing a cylindrical sensor array model using the k-Wave toolbox. The three-dimensional ultrasonic imaging system was employed simultaneously to accomplish the acquisition of three-dimensional experimental data for the breast model. Three-dimensional ultrasound image reconstruction of the digital phantom of breast tissue and the breast model was achieved by applying surface rendering and volume rendering algorithms to the acquired 3D data. The system exhibits excellent imaging resolution, as demonstrated by both simulation and experimental results. The complexity and time of signal acquisition in full 3D ultrasonic systems are effectively reduced by employing the 1 × 256 ring array to reconstruct 3D ultrasonic images. The proposed method also significantly reduces the complexity and computational burden of subsequent signal extraction and 3D reconstruction algorithms. The 3D ultrasonic imaging system exhibits characteristics of high resolution, high efficiency [29], and remarkable measurement accuracy.

## 2. Reconstruction Method for Three-Dimensional Ultrasound Images Based on the Sequences of Ultrasound Tomography

### 2.1. The Principles and Methods of the 3D Ultrasonic Image Reconstruction Algorithm Based on Surface Rendering

**Figure 1:**
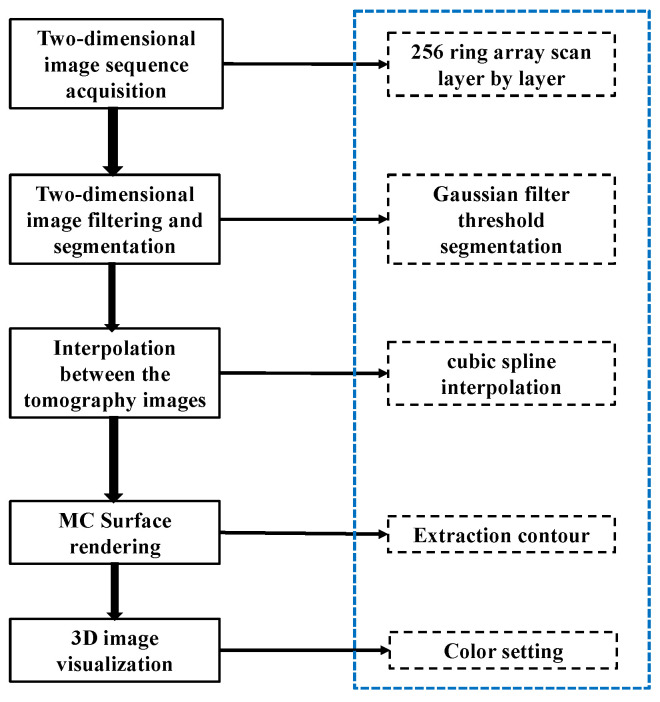
Surface rendering 3D reconstruction process.

The reconstruction of three-dimensional ultrasound imaging requires the extraction of information from a sequence of two-dimensional ultrasound images. The 32 sets of cross-sectional data collected with the three-dimensional ultrasound system at equal intervals provide essential volumetric information for the three-dimensional reconstruction of the breast model. The first step involved acquiring 32 sets of ultrasonic tomography image sequences using an improved B-mode imaging algorithm. The relative position and orientation of the two-dimensional image group are established within a three-dimensional space. The corresponding three-dimensional pixels are determined by the two-dimensional pixels, and the 3D volume data of the breast model is reconstructed [30]. The program flow of the 3D reconstruction and visualization algorithm, which is based on surface rendering, is illustrated in Figure 1. The algorithm effectively projects certain attributes from the original data onto a two-dimensional plane or surface, demonstrating notable advantages in terms of reduced data volume and accelerated rendering speed.

In order to enhance the reconstruction speed of three-dimensional ultrasonic images, it is necessary to preprocess ultrasonic tomography images for the construction of efficient volumetric data structures. The application of smoothing and segmentation techniques to sequences of ultrasonic tomography images can enhance the efficiency of traversing and resampling 3D volume data [31]. The Gaussian filter is employed for the purpose of noise and distortion reduction, functioning as an equivalent to a low-pass filter. Assume that the initial grayscale image *I*_0_ *= I*(*x*, *y*, 0), *I*(*x*, *y*, *t*) is the convolution of a two-dimensional Gaussian kernel

(1) $$G_{\sigma }=\frac{1}{2\pi \sigma^{2}}\exp\left(-\frac{\left|x^{2}\right|+\left|y^{2}\right|}{2\sigma^{2}}\right)$$

and *I*_0_,

(2) $$I\left(x,y,t\right)=G_{\sigma }*I_{0}$$

The image is smoothed and acquired at time *t*, where $t=0.5\sigma^{2}$.

The boundaries of breast tissue and tumors were extracted using threshold segmentation, owing to the significant disparity in gray values observed in ultrasonic tomography images. Feature extraction is an effective means of reducing the volume of data that requires processing. Relevant feature extraction is conducted to simplify the data effectively. The threshold segmentation method aims to effectively distinguish the foreground object from the background in an image [32]. A point (*x*, *y*) in the original image f(x,y) that satisfies f(x,y)≥T is referred to as an object point, while the remaining points are referred to as background points. The image obtained after threshold processing is defined as follows:

(3) $$g\left(x,y\right)=\left\{\begin{array}{l} 1,f\left(x,y\right)\geq T\\ 0,f\left(x,y\right)<T \end{array}\right.$$

The threshold *T* is determined according to a certain point on the trough in the gray histogram of the image [33].

To ensure the integrity and accuracy of the reconstructed 3D image, it is imperative to incorporate virtual slices between the segmented images. The cubic B-spline interpolation algorithm is employed in this study to enhance the resolution of 3D reconstructed images by performing interlayer interpolation. The existing tomography image can be denoted as *I_k−n_*, *I_k−(n−_*_1*)*_, …, *I_k−_*_1_, *I_k_*, *I_k+_*_1_, …, *I_k+(n−_*_1*)*_, *I_k+n_*, and the intermediate interpolated image is denoted as *I_k+d_*. Let the distances separating the two adjacent layers of pictures be 1, and let *d* represent the distance between the interpolated image *I_k+d_* and the known tomography image *I_k_*. The B-spline interpolation algorithm only requires convolving these discrete images with a finite-length continuous shock response *h*(*z*), which can be mathematically expressed by the formula,

(4) $$I_{k+d}=\sum_{m=-n}^{n}I_{k+m}\cdot h\left(m-d\right)$$

where,

(5) $$h_{3}\left(z\right)=\left\{\begin{array}{ll} \left(a+2\right){\left|z\right|}^{3}-\left(a+3\right){\left|z\right|}^{2}+1, & 0\leq \left|z\right|<1\\ a{\left|z\right|}^{3}-5a{\left|z\right|}^{2}+8a\left|z\right|-4a, & 1\leq \left|z\right|<2\\ 0, & others \end{array}\right.$$

Finally, MC (Marching Cubes) are employed for surface rendering. According to the determined threshold, the isoplane of reconstructed tissue is constructed. It can be expressed as

(6) $$\left\{\left(x,y,z\right)|f\left(x,y,z\right)=c\right\}$$

where *c* represents a constant. The voxel-by-voxel construction is performed by scanning two layers of data after reading the 3D discrete regular data field. The value of each voxel corner is compared to the given threshold value *c*, and the voxel’s index table is constructed based on the result of this comparison. According to the index table, the boundary voxels that intersect with the isosurface are identified. The intersection point between the voxel edge and the isoplane is determined using the method of linear interpolation. The normal vector at each voxel corner is obtained using the central difference method. The normal vector at each vertex of the triangular surface is obtained through the application of the linear interpolation method. The contour map is generated based on the coordinates and normal vectors of each vertex of the triangular surfaces. Lastly, the designated color scheme is applied to the organization and it is presented with a translucent effect.

### 2.2. The Principles and Methods of 3D Ultrasonic Image Reconstruction Algorithms Based on Volume Rendering

In contrast to surface rendering, volume rendering utilizes the entirety of volumetric data. The processing of each data point is conducted prior to the synthesis of the 3D image. The volume rendering algorithm enables the reconstruction of an image that preserves intricate details, effectively highlighting the distinctive features and hierarchical relationships in the data [34]. It has a good effect on the three-dimensional display of tissues and organs with ambiguous structural features (such as soft tissues and blood vessels, etc.).

**Figure 2:**
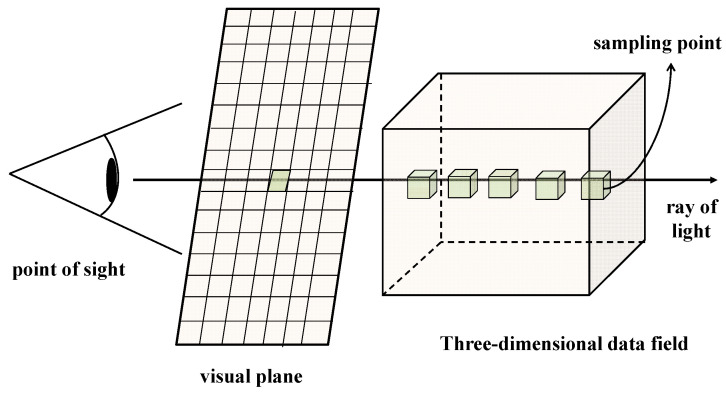
Schematic diagram of the ray-casting algorithm.

The ray-casting algorithm is a typical volume rendering reconstruction algorithm, characterized by its underlying principle: assuming that each data point in a 3D data field possesses both chromaticity and opacity attributes [35]. By employing the parallel projection technique, it is assumed that the viewpoint extends to infinity. Based on the current position of the viewpoint, a ray is emitted from each pixel of the projection plane through the three-dimensional data field [36]. The light is sampled at several equidistant points. The opacity and color values of these sampling points are computed using the trilinear interpolation method, as illustrated in Figure 2. The synthesis is then performed in a front-to-back order, based on the opacity value of each sampling point and its corresponding color value. The process ends when the light is completely absorbed or passes through the data field. Thus, the color value at that pixel on the screen can be obtained.

## 3. The Reconstruction of a Breast Phantom 3D Ultrasound Simulation Image Utilizing the 1 × 256 Ring Array

### 3.1. The Reconstruction and Resolution Evaluation of Ultrasonic Reflection Simulation Images

**Figure 3:**
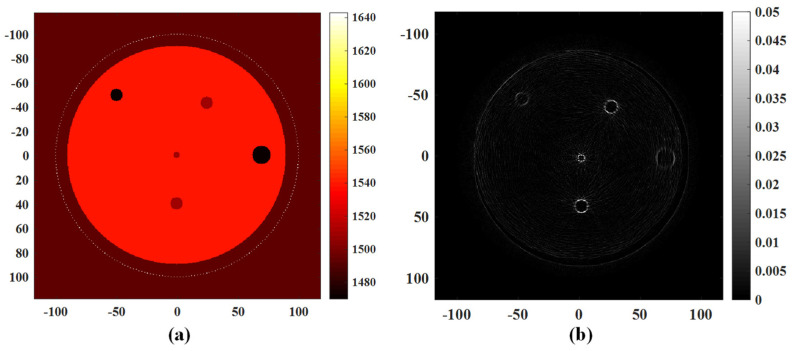
The simulation experiment of a tissue-mimicking ultrasound phantom for US-CT based on k-Wave toolbox. (**a**) Simulation model. (**b**) Simulation reconstruction result.

The volume dataset utilized for 3D reconstruction of the breast model in this study was acquired through a layer-by-layer scanning process utilizing a 256-ring array. The quality of the reconstructed ultrasonic tomography image directly impacts the efficacy of the 3D image reconstruction [37]. The time-domain simulation and signal acquisition of 256-ring sensor array sound waves were conducted using the k-Wave acoustic toolbox. The simulation verification of the improved B-mode imaging algorithm has been completed. The present study establishes a 256-ring array transducer with a diameter of 200 mm and develops a simulation model for breast tissue sections to accurately simulate the processes of ultrasonic emission and propagation within biological tissues, as well as echo signal reception and storage. The background medium is water, as depicted in Figure 3a. The sinusoidal pulse wave with a period of 5 is used as the excitation signal, while the incident signal exhibits a central frequency of 3 MHz. The grid step size of 0.3 mm satisfies the spatial sampling theorem as it is smaller than one-third of the wavelength. The time step is set to 0.1 μs, satisfying the Nyquist-Shannon sampling theorem. The value of the PML (Perfect Match Layer) is set to 20. The signals are transmitted sequentially by each array, while the remaining arrays receive the signals. The acquisition of ultrasonic signals using a ring array, based on a simulation model of breast tissue sections, was successfully accomplished.

The breast tissue section model consists of a background material that simulates adipose tissue and five targets that simulate lesions of varying degrees. Table 1 displays the size and acoustic parameters for each component of the established model.

The reflected signal necessary for image reconstruction is extracted from the collected simulation data, and the improved B-mode algorithm’s reconstruction results are presented in Figure 3b. According to the reconstruction results of the simulation model of a breast tissue section, the number, shape, size and position of the internal targets were basically consistent with the set theoretical values.

The imaging resolution is assessed through two metrics: the minimum detectable lesion size and the minimal discernible distance between two lesions. In order to investigate the resolution of ultrasonic tomography images, a multi-lesion simulation model was established with varying sizes (diameters of 0.3 mm, 0.5 mm, and 1 mm). The same simulation conditions were employed to establish a double lesion simulation model with an identical size (diameter of 1 mm), but varying intervals. The distance d between the two lesions progressively decreased in both the transverse and longitudinal directions, with values decreasing from 6 mm to 5 mm, 4 mm, 3 mm, 2 mm, and finally, reaching 1 mm.

**Figure 4:**
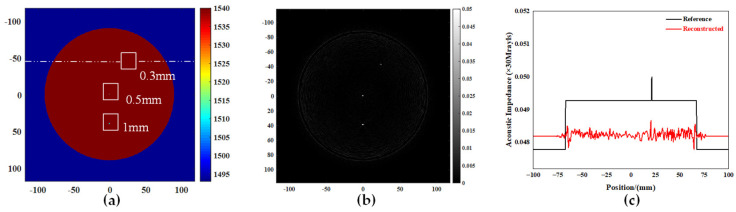
Simulation experiment of a multi-lesion model. (**a**) Multi-lesion simulation model. (**b**) Reconstructed image. (**c**) Quantification of the reconstructed image of a target with a diameter of 0.3 mm.

It is evident from Figure 4b that, under the given simulation conditions, the improved B-mode imaging algorithm can reconstruct a lesion with a diameter as small as 0.3 mm. The dashed line depicted in Figure 4a represents a one-dimensional transversal that passes through the central region of a circle with a diameter measuring 0.3 mm. The acoustic impedance value curve along the transversal line in the simulation model and the corresponding gray value curve along the transversal line in the reconstructed image are plotted on a shared coordinate system, as illustrated in Figure 4c. The quantitative estimation of the target’s size and position is derived from analyzing the corresponding inflection points in both curves, as well as considering the overall trend exhibited by these curves. Remarkably, the reconstructed dimensions and location of the 0.3 mm diameter target align closely with theoretical expectation.

**Figure 5:**
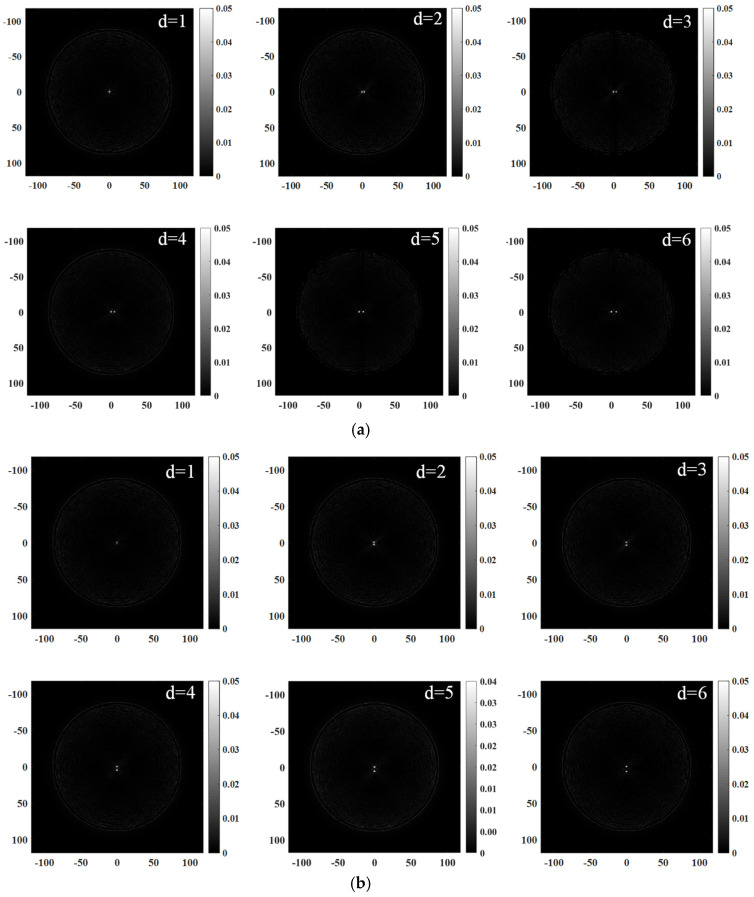
Reconstructed images of double lesions at different horizontal intervals d: (**a**) horizontal and (**b**) vertical.

**Figure 6:**
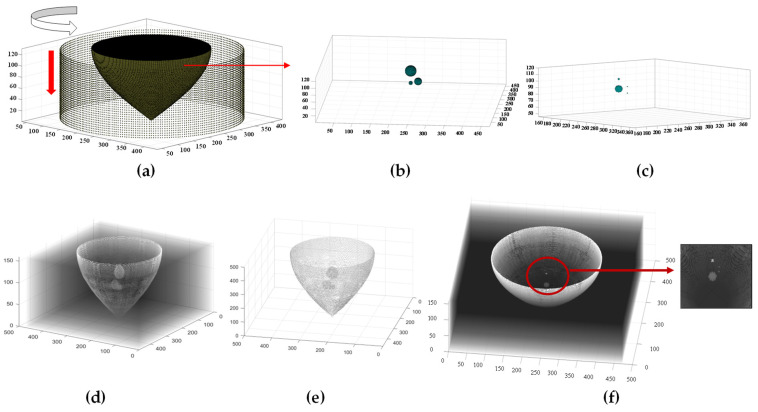
Three-dimensional time-domain simulation of acoustic wave propagation in a digital breast phantom using the k-Wave toolbox and 3D reconstruction results of the model. (**a**) 3D simulation model. (**b**) Lesion model 1. (**c**) Lesion model 2. (**d**) Three-dimensional reconstruction image of model 1 and visualization in a transparent manner. (**e**) Results of 3D reconstruction after segmentation. (**f**) Three-dimensional reconstruction image of model 2.

The simulated reconstruction image revealed that, as depicted in Figure 5 and Figure 6, reducing the lesion distance to 1 mm resulted in an incomplete separation between the two lesions. The imaging resolution of the two-lesion model was demonstrated to be 2 mm under these specific imaging conditions.

### 3.2. The Volume Rendering 3D Reconstruction of the Simulation Model

According to the operational principle of the three-dimensional ultrasound imaging system that we have developed, a 3D simulation model of cylindrical array-mammary-tumor was established using the k-Wave toolbox. The model consists of a 32-layer, 256-ring array as shown in Figure 6a. The two distinct lesion models are illustrated in Figure 6b,c. The dimensions of the three-dimensional space (water volume) measure 256 × 256 × 256 mm^3^, with a grid resolution set at 0.3 mm. The interlayer spacing of a 256-ring array measures 1.5 mm. The upper surface of the digital phantom breast tissue has a diameter measuring 160 mm. According to the resolution of the reconstructed simulation image obtained from mammary ultrasound reflection tomography, the diameters of the spherical lesions in the first simulation model were 15 mm, 10 mm, and 5 mm, respectively. In this model, a distance of 2 mm was set between tumor lesions with a diameter of 5 mm and those with a diameter of 10 mm. The spherical lesions in the second simulation model measured 5 mm, 1 mm, 0.3 mm, and 0.3 mm, respectively. The sound velocities of water, mammary tissue, and lesions were 1500 m/s, 1510 m/s, and 1560 m/s, respectively. The densities of water, mammary tissue, and lesions are 1000 kg/m^3^, 1040 kg/m^3^, and 1060 kg/m^3^, respectively. During the simulation, data from a 256-ring array is collected layer by layer, with each layer employing the transmitting and receiving mode of a 2D ring array.

The volume data collected is classified and extracted. The improved B-mode algorithm was employed for the reconstructing of 32-layer ultrasonic reflection tomography images in both models. After applying Gaussian filtering and threshold segmentation, the boundary information of breast tissue and lesions in the ultrasound reflection tomography simulation image becomes more distinct. The 3D reconstruction results of the two models, which were obtained through isosurface extraction using the Marching Cubes algorithm and visualized with a translucent effect, are presented in Figure 6. The 3D reconstruction results with 128 layers are illustrated in Figure 6d. The reconstructed results of the 3D ultrasonic image with 256 slices and segmentation are presented in Figure 6e. It can be observed that as the number of interpolation layers increases, the shape and size of each tissue in the reconstructed image become more similar to those in the simulation model. The three-dimensional lesion reconstruction size of this model can reach 0.3 mm, as depicted in Figure 6f.

**Figure 7:**
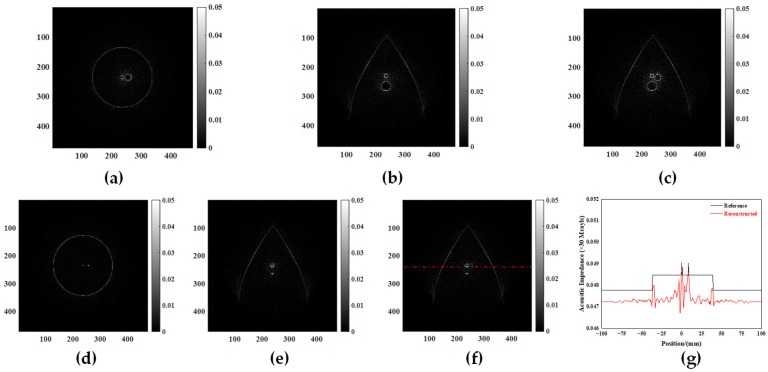
Typical sectional images of model 1 and model 2 in three directions. (**a**) Transverse of model 1. (**b**) Coronal of model 1. (**c**) Sagittal of model 1. (**d**) Transverse of model 2. (**e**) Coronal of model 2. (**f**) Sagittal of model 2. (**g**) Quantification of the reconstructed image of a target with a diameter of 0.3 mm.

In order to quantitatively evaluate the resolution of the 3D ultrasound reconstruction images of the breast digital phantom, the resolution quantization method of the 2D ultrasonic simulation reconstructed image is employed. The lamellar data were read based on the location of the 5 mm diameter target in simulation model 1. Coronal and sagittal ultrasound images of the digital breast phantom center were reconstructed, which also contained the spherical center of the lesion with a diameter of 5 mm, as shown in Figure 7. The complete separation of targets with diameters of 5 mm and 10 mm can be observed in Figure 7a,c. According to the results of ultrasound tomography reconstruction in the double lesion model, it is evident that the 3D simulation reconstructed image of the digital breast phantom exhibits a minimum interlesion distance of 2 mm.

Similarly, we extracted lamellar data from simulation model 2 based on the precise localization of the target with a diameter of 0.3 mm. Coronal and sagittal ultrasound images of the digital breast phantom center were reconstructed, as shown in Figure 7. The dashed line in Figure 7f is a horizontal line passing through the center of the target sphere with a diameter of 0.3 mm. The black curve in Figure 7g represents the acoustic impedance value corresponding to this profile in the simulation model, while the red curve depicts the grayscale value associated with this profile in the reconstructed image. The inflection points in the two curves consistently exhibit the same positions, while the overall trend of the curves remains congruent. In conjunction with the quantitative findings from ultrasonic tomography images of a multi-lesion model, our results demonstrate that digital phantom three-dimensional ultrasound imaging can accurately reconstruct lesions as small as 0.3 mm under simulated conditions.

### 3.3. The Volume Rendering 3D Reconstruction of the Simulation Model

The process of 3D reconstruction based on volume rendering requires the handling of extensive and intricate individual data, posing a challenge due to its magnitude and complexity. The existing image workstations lack sufficient computational power to generate reconstruction results of superior quality. The MITK (Medical Imaging Tool Kit v2022.04) algorithm platform is an integrated medical image algorithm and visualization platform, addressing the limitation of separating VTK and ITK algorithms and visualization by combining both seamlessly. Therefore, in this paper, the ray-casting algorithm was chosen to perform a three-dimensional reconstruction of the aforementioned breast simulation model based on the MITK v2022.04 platform.

The volume rendering model utilized in MITK v2022.04 is VolumeModel, which encompasses three class members: Volume provides access to the volume data; VolumeProperty allows adjustment of algorithm parameters; and VolumeRenderer handles the final rendering process. The process is as follows: (1) Generate the m_VolumeModel object that is required by the ray-casting algorithm for rendering. (2) By employing a series of Get and Set functions, the transfer function (comprising gray value—color transfer function, gray value—photometric transfer function, and gradient value—photometric transfer function) as well as light effect parameters can be established.

**Figure 8:**
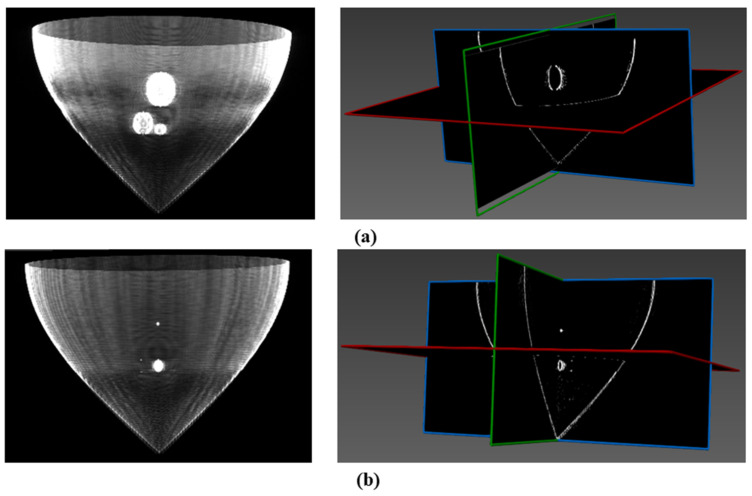
The 3D ultrasonic image reconstruction results are based on MITK v2022.04. Red represents the axial plane, green represents the sagittal plane, and blue represents the coronal plane. (**a**) Simulation model 1. (**b**) Simulation model 2.

Considering that MITK v2022.04 reads data in the DICOM (Digital Imaging and Communications in Medicine) format, we transformed the simulated tomography reconstruction images into a standard ultrasonic DICOM file sequence. The layer thickness was set to 1 mm and the layer spacing to 0.5 mm, which were imported into MITK v2022.04 for subsequent 3D reconstruction using the ray-casting method. The three-dimensional reconstruction effects of the two models are illustrated in Figure 8, showcasing the visualization of tumors within the digital breast phantom from multiple perspectives. Furthermore, employing this method also allows for the reconstruction of spherical tumors with a diameter as small as 0.3 mm.

## 4. The Reconstruction Experiment of Breast Three-Dimensional Ultrasound Based on the 1 × 256 Ring Array

### 4.1. The Test Imaging of a Tissue-Mimicking Ultrasound Phantom for US-CT

**Figure 9:**
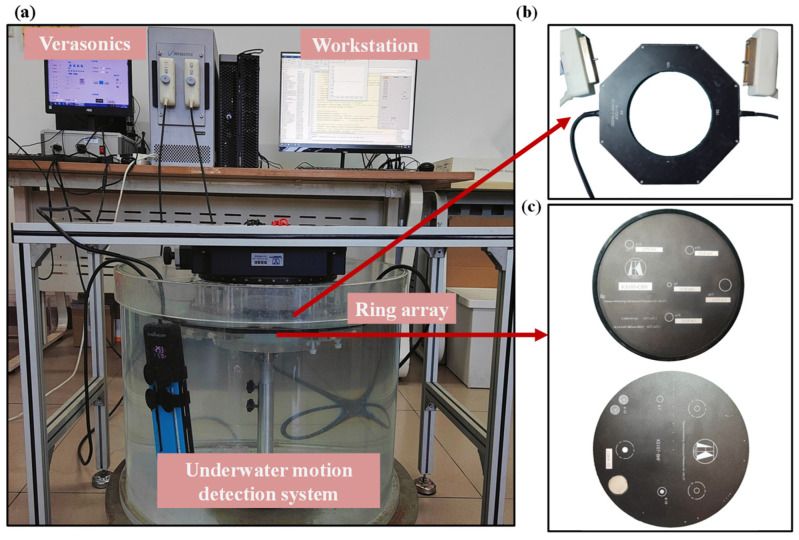
Experiment on ultrasonic reflection tomography. (**a**) Ultrasonic underwater imaging test system. (**b**) The 1 × 256 ring array. (**c**) Tissue-mimicking ultrasound phantom for US-CT.

In order to validate the congruity between experimental ultrasonic reflection tomography reconstruction results and simulation outcomes, the tissue-mimicking acoustic velocity detection phantom (KS105-CHV, Institute of Acoustics of the Chinese Academy of Sciences, Beijing, China) and the resolution ultrasonic phantom (KS107-BHF, Institute of Acoustics of the Chinese Academy of Sciences, Beijing, China), developed by the Institute of Acoustics, Chinese Academy of Sciences, were tested using a 256-piezoelectric ring array in this study. The experimental apparatus is depicted in Figure 9a. The ring array was securely attached by a fixed component and submerged in the water. The ultrasound phantom was precisely positioned at the center of the ring array, while maintaining a constant temperature of approximately 23 °C within the water tank using a heating rod. The 256-ring sensor array possesses a diameter of 20 cm, operates at a center frequency of 3 MHz, has an array height of 10 mm, an array spacer measuring 0.2 mm, and an array center distance of 2.454 mm, as shown in Figure 9b. The KS105-CHV Phantom is a cylindrical structure with a diameter of 180 mm. The interior contains cylindrical targets with three different diameters: 5 mm, 10 mm, and 15 mm. The sound velocity of the background material is (1540 ± 10) m/s, while that of the target is (1505 ± 10) m/s and (1477 ± 10) m/s, respectively. The targets are distributed in different locations within the phantom based on their varying diameters and sound velocity values. The KS107-BHF phantom is equipped with multiple target groups consisting of axial and lateral resolution lines, simulated tumor foci, and a single target line. In addition, there are three triangular-arranged support columns with sound absorption characteristics. The target line diameters are 1 mm and 0.3 mm. The simulated tumor lesion exhibited a diameter of 10 mm. The target lines within the target line group are progressively reduced from 4 mm to 1 mm at intervals of 1 mm, as shown in Figure 9c.

**Figure 10:**
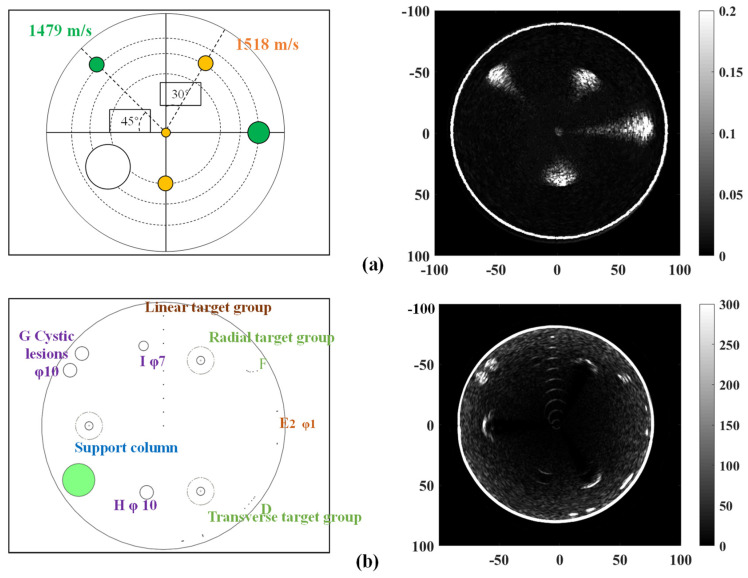
Reconstruction of the tissue-membrane ultrasound phantom using ultrasound reflection tomography. (**a**) Sound velocity detection. The orange target has a sound speed of 1518 m/s, while the green target has a sound speed of 1479 m/s. (**b**) Imaging resolution detection.

Figure 10 shows the ultrasonic reflection tomography results of the two phantoms. The reconstructed image of KS105-CHV reveals subtle disparities in the shape and size of the tumor when compared to their actual values. The texture of the target (which is characterized by solid features) in the reconstructed image exhibits homogeneity, while maintaining a distinct outline. The reconstructed image of KS107-BHF demonstrated that the location and size of the two tumor-like lesions G (with a diameter of 10 mm) were nearly consistent with their actual dimensions. The texture of both the target G (which is characterized by cystic features) and the background tissue in the reconstructed image shows a relatively uneven distribution. The reconstructed target contour displays a certain level of indistinctness. Due to the obstruction caused by the triangular-arranged support columns with sound absorption characteristics, the imaging results of lithiasis lesion I and cystic lesion H exhibited discontinuous arcs. The reconstruction results of linear target groups reveal that the size reconstruction error of target lines with identical diameters exhibits an increasing trend as the testing depth increases. The reconstruction error of the target line with a diameter of 1 mm is comparatively smaller than that of the target line with a diameter of 0.3 mm. The radial target group F can be clearly visualized at a distance of 3 mm, whereas it remains unresolvable at a distance of 2 mm. However, the reconstructed image of the transverse target line group cannot distinguish the targets. The experimental results demonstrate that the minimum achievable target size in ultrasonic tomography image reconstruction with a 256-piezoelectric ring array is 0.3 mm. However, the measurements of the reconstruction still deviate from the expected dimensions. The reconstructed size of the target line with a diameter of 1 mm exhibits excellent agreement with the actual value. The reconstructed image achieves a radial resolution of 3 mm.

### 4.2. Three-Dimensional Ultrasonic Reconstruction System

**Figure 11:**
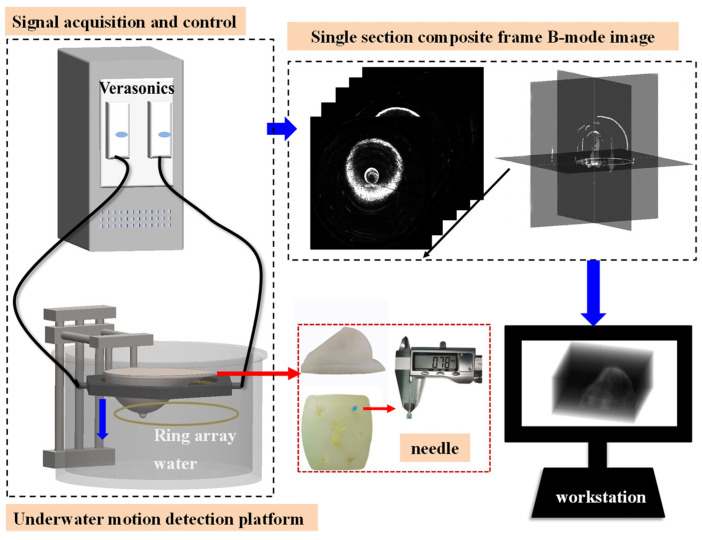
Diagram of the three-dimensional ultrasonic imaging system.

The three-dimensional ultrasonic imaging system, which is based on the 1 × 256 piezoelectric ring array as presented in this study, is depicted in Figure 11. The 3D ultrasound imaging system consists of a 256-piezoelectric ring array, a motion control platform, an underwater fixed platform, a data acquisition platform, and a computer workstation. The host computer precisely controls the 256-ring array to move uniformly in the vertical direction, enabling accurate cylindrical detection. The mobile platform exhibits a vertical displacement range of 70 mm, while simultaneously maintaining a positioning accuracy of 0.1 mm. In order to achieve a more realistic three-dimensional imaging effect resembling actual breast tissue, a customized breast-shaped model was employed. The material is composed of a proprietary gel and exhibits acoustic properties that are similar to those of authentic mammary tissue. The breast model has dimensions of 160 mm × 135 mm × 60 mm and contains six polyurethane rubber lumps with irregular shapes. The mass demonstrates a sound velocity of 1520 m/s. To assess the spatial resolution of the reconstructed image from the 3D ultrasound imaging system, a needle with a diameter of 0.78 mm was inserted at its base. The breast model was suspended at the center of the ring array during the test to accurately simulate the shape of a woman’s breast in the prone position. The Verasonics Vantage System executes a customized data acquisition script to control the 256-element ring array, enabling automatic electronic scanning as well as ultrasonic data acquisition and storage. The 256 elements in the ring array correspond to the 256 channels used for transmission and reception in the acquisition system. In the data acquisition script, set the AC voltage to a magnitude of 30 Vpp. The transmitted signal should be a sinusoidal pulse waveform encompassing 5 complete cycles, while ensuring a sampling frequency of 11.9 MHz. During the collection process, a single array element is utilized for signal transmission, while all other array elements receive signals, resulting in a total of 256 transmitted signals. The acquisition process involves capturing a total of 256 × 256 ultrasonic signals, with each frame of data taking approximately 0.14 s to collect. The 3D ultrasonic system acquires data at intervals of 1.5 mm, and the resulting 3D array, which is formed by combining data from 32 acquisition instances, is ultimately stored in the received data register of the workstation. The defined signal extraction method is then employed for data processing, enabling the extraction of the reflected signal necessary for 3D reconstruction. The 3D reconstruction algorithm for surface rendering was executed on the workstation, ultimately accomplishing the 3D ultrasound image reconstruction of the breast model.

### 4.3. Three-Dimensional Ultrasound Imaging of a Breast Model

**Figure 12:**
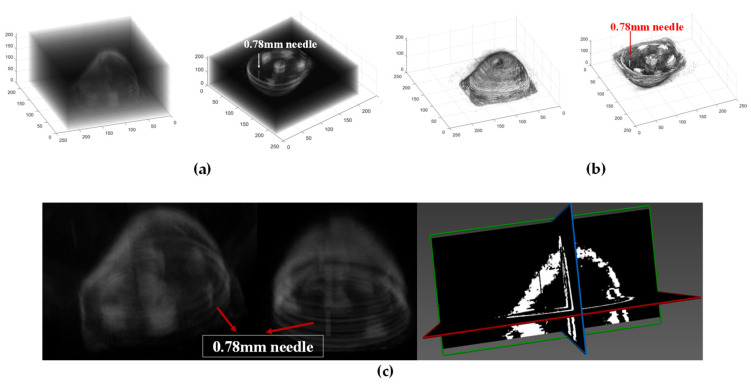
Three-dimensional reconstruction of the breast model. (**a**) Surface-rendered three-dimensional reconstruction of the breast model in translucent mode. (**b**) Surface-rendered three-dimensional reconstruction of the breast model in split mode. (**c**) Volume-rendered 3D reconstruction of the breast model.

During the testing process, it is essential to ensure continuous submersion of the ring array. The 256-ring array is controlled by a motion platform, enabling vertical movement of the ring array from the chest wall of the latex model to the nipple at a consistent velocity. The tomographic data were acquired at intervals of 1.5 mm, with a total of 32 acquisitions performed to obtain comprehensive three-dimensional volumetric data of the breast model. By employing the enhanced B-mode imaging algorithm, we successfully acquired a sequence of ultrasound reflection tomography images of a breast model. According to the surface rendering reconstruction algorithm, these image data undergo preprocessing, resulting in the acquisition of high-resolution 3D volume data for the breast model through interpolation. The 3D surface model of the breast model was constructed using the MC. The results of the 3D reconstruction are depicted in Figure 12a,b. The 3D reconstruction reveals a distinct delineation of the breast model and the irregular polyurethane mass, while providing clear visualization of the needle with a diameter measuring 0.78 mm.

The volume data of the breast model obtained through experimental testing was subjected to processing. The MITK v2022.04 platform was utilized to employ a ray projection method for the reconstruction of the breast model in 3D volume rendering. The 3D reconstruction results are depicted in Figure 12c. 

The reconstruction results reveal the abundant volumetric information contained within the 3D data field. The volume rendering algorithm produces a 3D image that enhances the visibility of both the internal masses within the breast model and the 0.78 mm needle. 

**Figure 13:**
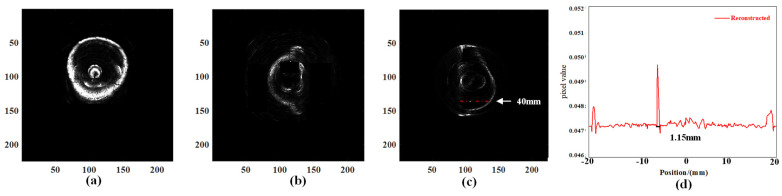
Typical sectional images of the breast model in three directions. (**a**) Transverse. (**b**) Coronal. (**c**) Sagittal. (**d**) Quantification of the reconstructed image of the needle with a diameter of 0.78 mm.

Based on the needle’s precise location, data from all layers were extracted and used to reconstruct a horizontal section of the 18th layer, as well as sagittal and coronal plane images at corresponding positions, as illustrated in Figure 13. The dashed line depicted in Figure 13c traverses the needle and measures a length of 40 mm. The curve represents the pixel values along the dashed line in the reconstructed image. The diameter of the reconstructed needle is approximately 1.15 mm, as determined by measuring the distance between the inflection points. The estimated absolute error in the reconstructed size is approximately 0.37 mm. This suggests that our imaging system and methodology still require continuous improvements.

The proposed 3D ultrasound imaging system was employed for data acquisition and the reconstruction of the breast model’s 3D ultrasound images, with an initial determination of its spatial resolution at approximately 0.78 mm. Previously, Liu et al. [38]. proposed a three-dimensional ultrasonic reconstruction system with the cylindrical motion of four piezoelectric micromachined ultrasonic transducer (PMUT) rotatable linear arrays. According to Table 2, this system demonstrates the capability to reconstruct lesions with a minimum size of approximately 10 mm. In contrast, the proposed 3D ultrasound reconstruction system significantly improves spatial resolution.

The needle utilized in this experiment deviates from the composition of the lesions found in actual breast tissue, resulting in a certain degree of disparity in acoustic impedance values between them. The reconstruction resolution of the 3D ultrasound imaging system can be preliminarily validated through experimental results obtained from needle-simulated lesions in this study. However, in practical application, the tissue architecture of breast lesions presents a more intricate composition that encompasses both cystic and solid components. This leads to an increased diversity in the mode of ultrasound propagation within the diseased tissue, potentially resulting in a slight reduction in imaging resolution and accuracy of lesion detection. Therefore, future investigations should prioritize enhancing the experimental protocol further and dedicating substantial efforts to optimizing image quality, refining the operational process, and integrating the system to augment the clinical relevance of this study.

## 5. Conclusions

The present study proposes a high-resolution 3D ultrasound imaging system, based on a 1 × 256 ring array, which holds potential for future application in breast cancer detection. The system successfully demonstrated its capability for 3D ultrasound reconstruction by reconstructing 3D ultrasound images of various breast models with diverse shapes and acoustic characteristics in the course of the study. The experimental findings presented in this paper further demonstrate that the spatial resolution of the 3D reconstructed image can achieve an approximate value of approximately 0.78 mm. The interlayer resolution of the ultrasonic tomography image is 1.5 mm. Simultaneously, based on the k-Wave toolbox, a simulation model of a cylindrical array is established to perform a 3D time-domain acoustic simulation of a digital breast phantom, in accordance with the system’s 3D ultrasonic imaging principle. The smallest lesion reconstructed from the simulation data had a diameter of 0.3 mm. The simulation results and experimental reconstruction demonstrate the system’s capability to achieve rapid and high-quality 3D image reconstruction of breast models. The complexity of 3D ultrasound imaging systems is significantly reduced in terms of data acquisition and storage, acquisition time, effective signal extraction, and corresponding 3D imaging algorithms. The high-resolution 3D ultrasound imaging system provides a viable technical solution for the practical implementation of breast 3D ultrasound imaging.

## Figures and Tables

**Table 1:** Parameters of breast tissue section model.

Component	Diameter (mm)	Sound Speed (m/s)	Density (kg/m^3^)
Background material	180	1515	1040
Target 1	10	1479	950
Target 2	10	1518	1060
Target 3	5	1518	1060
Target 4	15	1479	950
Target 5	10	1518	1060

**Table 2:** The research prototypes of 3D ultrasound reconstruction system towards the detection of breast cancer.

Three-Dimensional Ultrasound Imaging System	Liu et al. [38]	This Work
Primary sensing component	Four 1 × 128 PMUT linear arrays	1 × 256 piezoelectric ring array
Center frequency	3.5 MHz	3 MHz
Scanning mode	Rotate at an equal interval of 2°	Lift vertically at equal intervals of 1.5 mm
Spatial resolution	10 mm	0.78 mm

## Data Availability

Data are contained within the article.
